# Establishing the Thematic Framework for a Diabetes-Specific Health-Related Quality of Life Item Bank for Use in an English-Speaking Asian Population

**DOI:** 10.1371/journal.pone.0115654

**Published:** 2014-12-22

**Authors:** Odelia Koh, Jeannette Lee, Maudrene L. S. Tan, E-Shyong Tai, Ce Jin Foo, Kok Joon Chong, Su-Yen Goh, Yong Mong Bee, Julian Thumboo, Yin-Bun Cheung, Avjeet Singh, Hwee-Lin Wee

**Affiliations:** 1 Yong Loo Lin School of Medicine, National University of Singapore, Singapore, Singapore; 2 Saw Swee Hock School of Public Health, National University of Singapore, Singapore, Singapore; 3 Division of Endocrinology, Department of Medicine, National University Hospital, Singapore, Singapore; 4 Department of Medicine, Yong Loo Lin School of Medicine, National University of Singapore, Singapore, Singapore; 5 Department of Corporate Planning and Development, National University Hospital, Singapore, Singapore; 6 Department of Endocrinology, Singapore General Hospital, Singapore, Singapore; 7 Department of Rheumatology and Immunology, Singapore General Hospital, Singapore, Singapore; 8 Office of Clinical Sciences, Duke-NUS Graduate Medical School Singapore, Singapore, Singapore; 9 Centre for Quantitative Medicine, Duke-NUS Graduate Medical School Singapore, Singapore, Singapore; 10 International Health, University of Tampere, Tampere, Finland; 11 Department of Pharmacy, Faculty of Science, National University of Singapore, Singapore, Singapore; University of Calgary, Canada

## Abstract

**Aims:**

To establish a thematic framework for a Diabetes Mellitus (DM)-specific health-related quality of life (HRQoL) item bank by identifying important HRQoL themes and content gaps in existing DM-specific HRQoL measures and determining whether Patient-Reported Outcomes Measurement Information System (PROMIS) item banks are useful as a starting point.

**Methodology:**

English-speaking Type 2 DM patients were recruited from an outpatient specialist clinic in Singapore. Thematic analysis was performed through open coding and axial coding. Items from four existing DM-specific measures and PROMIS Version 1.0 and 2.0 item banks were compared with identified themes and sub-themes.

**Results:**

42 patients participated (25 men and 17 women; 28 Chinese, 4 Malay, 8 Indians, 2 other ethnicities). Median age was 53.70 years (IQR45.82–56.97) and the median disease duration was 11.13 (SD9.77) years. 10 subthemes (neutral emotions, coping emotions, empowered to help others, support from family, spend more time with family, relationships, financial burden on family, improved relationship, social support and religion/spirituality) were not covered by existing DM-specific measures. PROMIS covered 5 of 6 themes, 15 of 30 subthemes and 19 of 35 codes identified. Emotional distress (frustration, fear and anxiety) was most frequently mentioned (200 times).

**Conclusions:**

We had developed a thematic framework for assessing DM-specific HRQoL in a multi-ethnic Asian population, identified new items that needed to be written and confirmed that PROMIS was a useful starting point. We hope that better understanding and measurement of HRQoL of Asian DM patients will translate to better quality of care for them.

## Introduction

Diabetes mellitus (DM) is a major public health concern that imparts a significant burden to the individual and society. The global incidence of type 2 DM (T2DM) is rising at an alarming rate and is projected to affect 380 million in 2025 [1]. Asia will be at the forefront of this epidemic with the world's two most populous countries, namely China and India expected to have the greatest increase [2].

Traditionally, clinical outcomes had been solely measured by clinical and physiological variables like HbA1c. However, it has been shown that psychosocial factors are stronger predictors of morbidity and mortality [3]. This is because T2DM has a considerable impact on key aspects of patients' lives – physical, psychological, and social [4], affecting their health-related quality of life (HRQoL) [5], [6]. This phenomenon is generally attributed to the symptoms and complications of the disease, and its restrictive management, which requires lifestyle modification and pharmacotherapy [4], [7]. Because T2DM affects many areas of HRQoL, one of the challenges in understanding the disease impact is in identifying HRQoL themes that are relevant and important to these patients.

DM-specific HRQoL measures have been developed to address this need. These measures incorporate additional themes relevant to T2DM patients that are not addressed in generic measures, such as the Short Form 36 (SF-36) questionnaires. These include DM-specific symptoms such as neuropathic pain and poor vision due to diabetic retinopathy [8]. Some DM-specific HRQoL instruments are the Diabetes Quality of Life Measure (DQoL) [9], Audit of Diabetes Dependent QoL (ADDQoL) [10], Diabetes Health Profile (DHP) [11] and Diabetes-39 (D-39) [8].

The state-of-the art advancement in HRQoL measurement is the use of item banking, computerised adaptive testing (CAT) and item response theory (IRT)-derived short form questionnaires. CAT offers several advantages over static questionnaires: 1) it tailors the test to the patient whose latent trait is being measured. The tailored items lead to higher sensitivity and smaller variability in scores, hence, more accurate estimates, resulting in smaller sample size required for clinical trials. This translates to cost savings as fewer patients need to be recruited and the study can be completed in a shorter period of time; 2) it often requires fewer questions to arrive at an accurate estimate. Hence, it takes less time to administer the survey. 3) It allows the comparison of the latent trait between patients without having to ask the same set of questions as long as the questions come from the same item bank. Thus, this permits the contents of the survey to be targeted at individual patients and increases the relevance of the questions to the patients; 4) it reduces the problem of floor and ceiling effects in data collection [12].

The objective of this study was to establish a thematic framework for a T2DM- and cultural-specific HRQoL item bank by 1) identifying HRQoL themes of importance to Asian T2DM patients, 2) identifying content gaps in existing DM-specific HRQoL measures and 3) determining whether PROMIS item banks could serve as a core set of questions for the assessment of HRQoL among Asian T2DM patients.

## Materials and Methods

### Subjects

In this SingHealth Review Board-approved study (CIRB Ref No.: 2008/188/A), T2DM patients were recruited from an outpatient clinic at the Singapore General Hospital (SGH). Inclusion criteria for the study were 1) a diagnosis of T2DM made by an Endocrinologist, 2) aged 21 to 65 years, 3) able to read and understand English, 4) able to speak coherently and provide written informed consent to the SingHealth Review Board and 5) able to travel to the interview venue without assistance. Patients were stratified according to gender because of the anticipated sensitivity of certain issues that may be gender specific. To ensure homogeneity, participants were selected for each session based on their availability and characteristics, such as age and ethnicity. Recruitment was on a rolling basis and focus group sessions were convened whenever sufficient number of patients was available for a session.

Our aim was to recruit 4 to 7 participants for each session. The ideal number for focus group participation was 4 to 12 participants, with the optimal number dependent on the aim of the research study [13]. Participants were given a small token to cover transportation expenses. The interviews were structured as a moderator-led 2-hour open-ended discussion on T2DM-related HRQoL. All focus groups, interviews and subsequent data analysis were conducted in English. Participants also completed a questionnaire on background information.

The discussions began with the most general questions and participants were allowed to freely express their views. Moderator probes were minimized, with more specific questions only introduced subsequently to ensure sufficient breadth and depth were covered. The opening question for discussion was, “How has diabetes affected your health related quality of life?” Subsequent questions focused on three broad categories of 1) physical, 2) social well-being, and 3) emotional which are in accordance with the World Health Organization's (WHO) definition of health which is “a state of physical, mental and social well-being” [14].

### Data analysis

Focus group and interview discussions were voice-recorded, transcribed verbatim and analyzed using thematic analysis. The open-ended discussion guide and data-driven analytic methods adopted in this study were based on elements of the grounded theory to encourage development of conceptual frameworks derived from participant input, rather than existing concepts [15]. During thematic analysis, segments of texts describing similar manifestations were grouped into themes [16]. 5 independent analysts, 2 trained graduate students, a research assistant and 2 undergraduate students who were unfamiliar with existing frameworks of HRQoL, read the transcripts reflectively to identify relevant categories and assigned codes to text segments, in a process known as open coding [17]. Open coding is a data-driven process that identifies themes from data directly, rather than use ideas from existing literature or frameworks [18]. Codes were categorized as i) descriptive of HRQoL and ii) not descriptive of HRQoL but affects HRQoL (e.g. symptoms). Codes that represented a similar concept were grouped together to generate subthemes. Subthemes were further collapsed into themes, which were then organized into broad categories.

A codebook comprising five fields (code title, code definition, examples of use, guidelines for use, and relationship to other codes) was created as analysts coded sub-sets of transcripts in an unspecified number of rounds. This iterative process of independent coding and consensus meetings continued until investigators were satisfied that the codes could be consistently applied. The finalized codes were then independently applied to all transcripts and coding discrepancies were resolved at a final consensus meeting. This meant that each transcript was coded at least twice by at least two different coders, minimizing the chance of missing out important codes. The final output of thematic analysis was a list of descriptive HRQoL themes as discussed by study participants. Once the codebook was finalized, the focus group transcripts were recoded using NVivo 10 and the frequency at which the codes appeared in the transcripts were tabulated.

### Identifying of themes and subthemes to DM-specific instruments and PROMIS v1.0 Item Banks

Existing DM-specific instruments were identified through a systemic literature search using PubMed with the terms “diabetes mellitus” AND “quality of life”. Four widely used DM-specific instruments were identified from the search. They were DQoL, ADDQoL, DHP and D-39. Subthemes identified from the focus groups were compared against the items from these questionnaires. Items from 16 out of 21 PROMIS Version 1.0 and 2.0 Item Banks (excluding Global Health) were also compared with focus group subthemes. A content gap was identified when themes and subthemes elicited from focus groups were not addressed by any item from these existing sources.

## Results

### Focus group participants

42 patients with T2DM, age 21 to 65 participated in 18 focus groups conducted from Jan 2010 to February 2013. 18 sessions, each comprising of 1 to 4 participants, were conducted. Saturation of topics occurred at the 13th session. Majority of participants were Chinese (67%). Median age was 53.70 years (interquartile range 45.82–56.97) and median disease duration was 11.13 (SD 9.77) years (interquartile range of 4–15 years). Most participants were working (59%). Educational levels of participants were well varied among degree-holders (34%), post-secondary (31%), secondary education (21%) and below (14%).

### Thematic analysis

A total of 83 open codes were created, with 69 codes descriptive of HRQoL, 7 codes not descriptive of HRQoL and 7 codes labeled as broad category codes. Codes not descriptive of HRQoL included symptoms such as polyuria, polydipsia, weakness, fatigue, neutral emotions and coping emotions. Codes descriptive of HRQoL were further refined into 6 themes, 30 subthemes and 35 codes during axial coding. The 7 broad category codes were “Emotions”, “Empowerment”, “Family”, “Financial concerns”, “Management of T2DM”, and “Social”. The top 10 most frequently mentioned codes were “Change in dietary habits”, “Diet restriction”, “Direct medical and non-medical cost”, “Frustration”, “Fear”, “Lifestyle modifications”, “Support from family”, “Anxiety”, “Adapting to T2DM is challenging” and “Acceptance” (Table 1).

**Table 1:** Top 10 most frequently mentioned codes.

Rank	Code	Frequency of mention	Sample quotation
1	Change in dietary habits	118	“You buy those ah ricola and all these things, go for those sugar-free instead of taking the normal. yah yesh”
2	Diet restriction	107	“Well I love durians! But I cut off durians.”
3	Direct medical and non-medical cost	81	“Diabetics food are all very expensive” “You know have to prick your finger, (which) I find it very troublesome. And it actually rather costly, and I feel like I'm throwing money away. Because each strip, it's not cheap you know.”
4	Frustration	73	“But currently I maintain it (HbA1c) at 10. But the doctor say, if you maintain at 10 in, 5 years' time, you going to get stroke and all. That's why we feel very frustrated.”
5	Fear	70	“But it did scare me, like oh my god, if this happen will my child have diabetes then probably I wouldn't want to get married anymore.”
6	Lifestyle modifications	62	“But before I was diabetic, I'm quite lazy, I won't do exercise and all that. Everywhere I go I will take bus, even one stop also. But nowadays it's not like that. My lifestyle has changed a lot. I'm staying at the ninth floor, so I go up my steps all the way.”
7	Support from family	59	“My elder daughter is quite supportive of over these matters so lor. Now I go for all these (check ups), she's she's always there.”
8	Anxiety	57	“Of course, I am worries every day, I am worries the whole day.”
9	Adapting to DM is challenging	49	“I started exercising and I went on a diet so to drop my HbA1c to 6.5. I am along the way but it is still very difficult to maintain discipline.”
10	Acceptance	48	“I mean it's already there you cannot change you know, so why give yourself that kind of stress you know. Just take it (as it comes)”.

#### Themes/Subthemes/Codes addressed by existing DM-specific instruments

DM-specific instruments provided fairly good coverage of the subthemes (20 of 30) generated by the focus groups (Table 2). The 10 subthemes that were not addressed included neutral emotions, coping emotions, empowered to help others, support from family, spend more time with family, relationships, financial burden on family, improved relationship, social support and religion/spirituality. Subthemes under “Family” were somewhat addressed by D-39 and DQoL, with the exception of “support from family”, “spend more time with family” and “relationships”.

**Table 2:** Themes and Subthemes covered by existing DM-specific instruments and PROMIS version 1.0 and 2.0 item banks.

	ADDQoL	D-39	DHP-1	DQoL	PROMIS
EMOTIONS					
**Negative emotions**					
Frustration*			+		+
*e.g. I feel frustrated*					
Quick/hot-tempered*			+		+
*e.g. I am hot-tempered*					
Annoyance*	+		+		+
*e.g. I feel annoyed when others fuss over me*					
Depressed*		+	+		+
*e.g. I feel depressed*					
Hopeless*					+
*e.g. I feel hopeless*					
Helpless*			+		+
*e.g. I feel helpless*					
Disappointment*					+
*e.g. I feel disappointed*					
Anxiety*	+	+	+	+	+
*e.g. I feel anxious*					
Fear*			+		+
*e.g. I feel fearful*					
Deprived*			+		+
*e.g. My condition has prevented me from doing things that I want to do*					
Violence			+		
*e.g. I turn to violence as a response*					
Depression					
*e.g. I was diagnosed with depression*					
Embarrassed		+		+	
*e.g. I feel embarrassed*					
Mood swings			+		
*e.g. I have mood swings*					
**Positive emotions**					
Positivity when DM control is good			+	+	
*e.g. I feel positive when the control of my condition is good*					
**Neutral emotions**					
Nonchalant					
*e.g. I feel nonchalant towards my condition*					
**Coping emotions**					
Envy*					+
*e.g. I feel envious*					
Resigned*					+
*e.g. I feel resigned to my condition*					
Gratitude*					+
*e.g. I feel more thankful with what I have*					
Acceptance*					+
*e.g. I am comfortable with who I am*					
EMPOWERMENT					
**Empowered to help others**					
*e.g. I feel empowered to help others*					+
FAMILY					
Support from family*					
*e.g. I get support from my family*					+
Spend more time with family*					
*e.g. I spend more time with my family*					+
Family negatively affected by patient's DM*					
*e.g. My family has been negatively affected by my condition*		+		+	
Relationships*					
Strained relationships – spousal*					+
*e.g. DM has strained my relationship with my spouse*					
Strained relationships – children*					+
*e.g. DM has strained my relationship with my children*					
Strained relationships – other family members*					+
*e.g. DM has strained my relationship with my other family members*					
Improved relationships*					
*e.g. DM has improved my relationship with my family*					+
**Family planning**					
*e.g. DM has influenced my decision to have children*				+	
FINANCE					
**Opportunity cost of DM**					
*e.g. I have lost income because of my condition*	+				
**Direct medical and non-medical cost**					
*e.g. I have spent a lot of money on treating DM*		+			
**Financial burden on family**					
*e.g. My condition has increased the financial burden on my family*					
**Personal financial stress**					
*e.g. I experience personal financial stress because of my condition*		+			
DISEASE MANAGEMENT					
**Disease adaptation**					
Adapting to DM is challenging		+	+	+	
*e.g. I feel that adapting to my condition is challenging*					
Lifestyle modifications		+			
*e.g. I have to change my lifestyle*					
Work life modifications	+			+	
*e.g. I have to change my work life*					
**Dietary**					
Diet restriction	+	+	+	+	
*e.g. I cannot eat certain foods*					
Drink restriction	+	+	+		
*e.g. I cannot drink certain drinks*					
Change in dietary habits		+	+		
*e.g. I have made changes to my eating habits*					
Travel restriction*					
*e.g. My travel is limited*	+			+	+
Difficulties walking*					
e.g. I have difficulties walking		+			+
Reduced ability to do household chores*					
*e.g. I cannot do household chores*		+		+	+
Improved physical health status*					
*e.g. My physical health has improved*	+				+
Sleep*					
*My sleep is affected*		+		+	+
SOCIAL					
Do not want others to know*					
*e.g. I do not want others to know that I have DM*				+	+
See no need for others to know*					
*e.g. I do not think it is necessary for others to know that I have DM*				+	
Social support*					
*e.g. I have support from my friends*					+
Self-esteem*					
*e.g. I feel inferior to others*	+		+	+	+
Stigma and discrimination*					
**Discrimination perceived**	+			+	+
*e.g. I feel that others discriminate against me*					
**Lack of understanding by others**				+	+
*e.g. I feel that others do not understand me*					
**Social activities**					
**Changed participation in social activities because of DM**				+	
*e.g. I have to modify my participation in social activities*					
**Involved friends in social activities that were modified because of DM**					
*e.g. I have engaged my friends in my modified social activities*					
**Self-image**					
*e.g. I am conscious of how others view me*	+	+			
**Religion/spirituality**					
**Use religion to cope with DM**					
*e.g. I turn to religion to help me cope*					
**Reduced participation in religious activities**					
*e.g. I have to decrease my participation in religious activities*					

Subthemes under “Emotion” were mostly covered by DHP, with ADDQoL, D-39 and DQoL. Subthemes within “Physical impact due to T2DM” were well-covered by ADDQoL, D-39 and DQoL. The subthemes “Dietary” and “Disease adaptation” were comprehensively evaluated by ADDQoL, D-39, DHP and DQoL. Subthemes under “Social” were somewhat covered by ADDQoL, DHP and DQoL.

#### Themes/Subthemes/Codes addressed by PROMIS v1.0 and 2.0

PROMIS also provided fairly good coverage, addressing almost half of the generated codes (5 of 6 themes, 15 of 30 subthemes and 19 of 35 codes). PROMIS covered 6 out of 10 subthemes (coping emotions, empowered to help others, support from family, spend more time with family, relationships and improved relationships) and 7 out of 11 codes (envy, resigned, gratitude, acceptance, strained relationships—spousal, strained relationships—children, strained relationships—other family members) that were not covered by existing DM-specific instruments (Table 2).

Codes grouped under “Physical impact due to T2DM” (travel restriction, difficulties walking, reduced ability to do household chores, improved physical health status, sleep and independence), “Social” (do not want others to know, social support, self-esteem, and stigma and discrimination) and “Emotions” (negative emotions, coping emotions) were generally well addressed by DM-specific instruments and relevant PROMIS item banks. Of particular significance, all the codes under the subtheme “Relationships” (under the theme “Family”) were covered exclusively by PROMIS, but not any DM-specific instrument. Similarly, all 4 codes (envy, resigned, gratitude and acceptance) under the subtheme of “Coping emotions” were addressed only by PROMIS.

#### Themes/Subthemes/Codes not addressed by PROMIS v1.0 and 2.0

All the subthemes under “Management of T2DM” were not covered by PROMIS. Themes comprising of “Financial concerns” were not addressed by PROMIS and were only briefly covered by 3 DM-specific instruments. Codes under the theme “Social”, namely social activity, self-image and religion/spirituality were not covered by PROMIS. Out of these 3 themes, only half of the subthemes were addressed by DM-specific instruments.

#### Themes/Subthemes/Codes not addressed by both DM-specific instruments and PROMIS v1.0 and 2.0

4 subthemes (financial burden on family, religion/spirituality, neutral emotions, empowered to help others) were not addressed by both DM-specific instruments and PROMIS v1.0 and 2.0.

## Discussion

Through this qualitative study, we have established a thematic framework for developing DM-specific and cultural-specific HRQoL item bank that will be relevant to multi-ethnic Asian T2DM patients in Singapore, Asian countries with similar ethnic makeup and possibly in other Asian migrant communities overseas. In addition, we have identified content gaps from existing DM-specific instruments where new items need to be written. We have also confirmed that PROMIS Version 1.0 and 2.0 item banks serve as a useful starting point for the development of this DM-specific item bank. This is important as it means not re-inventing the wheel, thus, saving time and cost. More importantly, it allows for comparison with other studies using the same PROMIS item banks.

While “Change in dietary habits” and “Diet restriction” were the top two individual codes of importance to Asian T2DM patients (based on the frequency of mentions during the focus groups), we noticed that “Frustration”, “Fear” and “Anxiety” were mentioned 73, 70 and 57 times, respectively. These emotional subthemes were freely offered by the participants, even when the moderator did not prompt them to talk to about their emotional health, *‘lately I'm quite worried because I'm going for my knee cap transplant… I'm quite nervous and, a bit scared at it lah. Besides that, cos the doctor told me that, you know it may have blood clots. If I have blood clots I'll be in serious trouble…so I'm quite worried about this lah, so I really controlling my diabetic.’* Based on the definitions of the PROMIS network, emotional distress is characterized by 3 subthemes, namely “Anxiety”, “Depression” and “Anger” [19]. If taken together, emotional distress (“Frustration”, “Fear” and “Anxiety” mentioned 200 times) would have been the top most concern among this group of Singapore patients with T2DM. This finding points to the need for future research to develop an emotional distress item bank to cater for Asian patients with T2DM.

10 subthemes (neutral emotions, coping emotions, empowered to help others, relationships, financial burden on family, support from family, spend more time with family, improved relationship, social support, religion/spirituality) were not addressed by DM-specific instruments and we hypothesize that this is due to the presence of cultural differences in what constitutes HRQoL. Themes such as “Family” and “Religion/spirituality” were often referred to by our Asian participants, suggesting that these may be more relevant in the Asian socio-cultural context than in the West. For example, “Support from family”, a subtheme of “Family”, was the 6^th^ most talked about topic by the focus group participants. This was also noted by Kong et al [20] where “Family life” was rated as most important when the ADDQoL was administered to T2DM patients in China. “Religion/spirituality” appeared to be especially important in Asian societies, suggesting that Asian patients were more at ease with discussing issues related to religion and/or spirituality, a topic which could be a taboo in certain cultures. In many instances, focus group participants mentioned turning to religion for comfort such as *‘I always tell myself, if Lord has chosen me to be in His holy arms, I mean that's the time he'll ask me to go’,* as well as the impact of T2DM on their religious life—‘*I like to read books, especially the religious book (Quran). Now I find… very hard to concentrate because I can get tired easily.’* Similar subthemes of religion have been identified in previous qualitative studies [21]. Social activities, an aspect of HRQoL, may also be deemed more important in an Asian population. The study by Cheng et al [22] to develop a HRQoL measure relevant to Chinese T2DM patients in Canada, had to include additional themes such as “social interactions centred around meals” to the DQOL to make it more culturally appropriate.

Items from existing PROMIS item banks generally addressed the corresponding themes well. Hence, PROMIS item banks can serve as a useful core set of items for assessing HRQoL in T2DM patients with the following revisions: 1) Inclusion of additional themes. Patients with T2DM are constantly faced with demanding management of the disease; hence it is important to include themes such as “Coping emotions”, to ensure a more holistic measurement of HRQoL. Another important theme that should be included is “Financial concerns”. Finance is the key driving force behind many healthcare decisions. Unlike the healthcare financing system in the United States and United Kingdom, the health care financing philosophy in Singapore emphasizes individual responsibility and, thus, an essential co-payment by patients for all health services consumed. Hence, the financial burden posed by T2DM can be tremendous. Several countries in the region (e.g. Thailand, Indonesia and Malaysia) have similar co-payment schemes. As such, a financial item bank can be applicable to these other countries and will be a useful step forward as PROMIS expands its international outreach; 2) Inclusion of positive impact of T2DM. Currently, apart from the ADDQoL which uses a scale that spans both positives and negatives aspects of T2DM, other DM-specific instruments fail to measure the positive impact of T2DM on HRQoL. For some patients, the experience of being diagnosed with diabetes gave them the opportunity to adopt a ‘healthier’ lifestyle, which made them feel more confident and ‘healthier’ [23]. Thus, the positive impact of diabetes on daily activities and lifestyle should not be overlooked in the measurement of HRQoL.

Certain study limitations may preclude an exhaustive coverage of topics for all subgroups of T2DM patients, though it should be noted that as with qualitative research, focus group findings are not meant to be readily generalizable. First, participants were recruited from outpatient clinics, ambulatory and not stratified by disease characteristics, such as duration or specific organ involvement. As such, themes or subthemes that were potentially unique to inpatients or the more severely ill might have been missed. This was despite the fact that focus group participants recounted their experiences with hospitalizations. Second, it might be opined that concepts on family support, relationship issues, financial concerns or religiosity are not relevant to disease-specific instruments. However, as we had set up the discussion around “what are the issues that affect you physically, mentally and socially as a result of diabetes”, it would be reasonable to believe that from the patients' perspective, these constitute the concept of disease-specific HRQoL. The World Health Organization Quality of Life Questionnaire, for instance, also incorporated a question on financial burden. Given that there are significant costs associated with diabetes care, such as paying for expensive test strips, use of multiple medications, etc., it would be hard to imagine that financial concerns are not attributable to the disease per se. Third, the size of our focus groups tended to be smaller than expected (with 1-4 participants). This was largely due to last minute withdrawal from participants and the difficulty of scheduling a common time between them. Finally, PROMIS is an ongoing initiative with an evolving thematic framework; hence newly developed banks addressing the identified content gaps may be available by the time of study publication. Readers are advised to refer to the PROMIS website for the latest versions of the available item banks.

## Conclusions

In conclusion, pre-existing diabetes specific HRQOL instruments may not sufficiently address the concerns of Asian T2DM patients living in Singapore, the region or overseas. Given the increasing disease burden in Asia, further work is required to develop an item bank that more accurately and more precisely captures HRQoL of Asian T2DM patients so that these tools can be employed in routine clinical practice and interventions designed to improve the HRQoL of patients.
